# VLSI Structures for DNA Sequencing—A Survey

**DOI:** 10.3390/bioengineering7020049

**Published:** 2020-05-31

**Authors:** Mohammad A. Islam, Palash K. Datta, Harley Myler

**Affiliations:** Phillip M. Drayer Department of Electrical Engineering, Lamar University, Beaumont, TX 77705, USA; mislam3@lamar.edu (M.A.I.); pdatta@lamar.edu (P.K.D.)

**Keywords:** DNA sequencing, DNA hybridization, DNA sensor, GAA nanowire MOSFET

## Abstract

DNA sequencing is a critical functionality in biomedical research, and technical advances that improve it have important implications for human health. Novel methods by which sequencing can be accomplished in more accurate, high-throughput, and faster ways are in development. Here, we review VLSI biosensors for nucleotide detection and DNA sequencing. Implementation strategies are discussed and split into function-specific architectures that are presented for reported design examples from the literature. Lastly, we briefly introduce a new approach to sequencing using Gate All-Around (GAA) nanowire Metal Oxide Semiconductor Field Effect Transistors (MOSFETs) that has significant implications for the field.

## 1. Introduction

**Figure 1:**
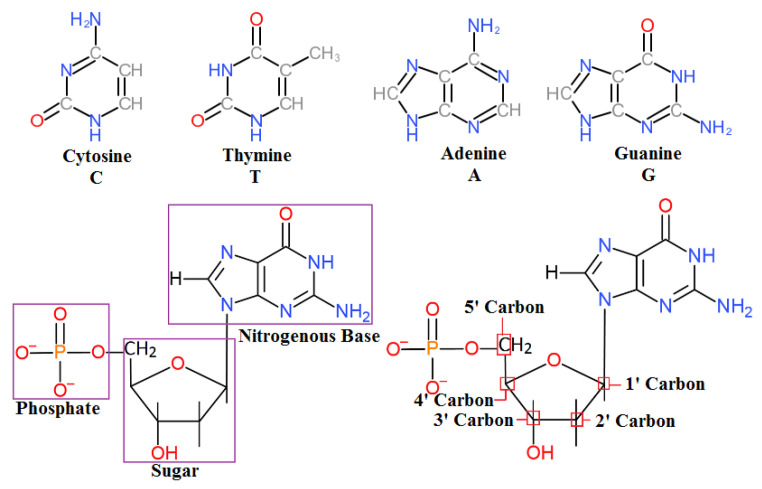
Four types of nitrogenous base and structure of a single nucleotide.

Nucleic acids are the biomolecules that are essential for the continuity of all living organisms and store the hereditary information that passes from one generation to the next. This code for life is stored as deoxyribonucleic acid (DNA). DNA is composed of four basic nucleotides composed of a phosphate group, a sugar, and one of four different nitrogenous bases (Figure 1).

The overall structure of DNA is negatively charged and has a negative electrostatic potential due to a negatively charged phosphate backbone [1,2,3]. To know more about DNA’s structures and its molecular charge, readers are requested to consult [4,5,6]. Nucleotides are electroactive compounds that produce reduction and oxidation signals after hybridization [7]. These high electrostatic potentials can be exploited with VLSI charge-sensitive electronic structures. The sequence of DNA is encoded within each nitrogenous base: adenine (A), guanine (G), cytosine (C), or thymine (T), and this 4-bit system allows for large amounts of information to be stored in a single DNA molecule, which may contain upward of 3 billion bases. Reading this code from the DNA has provided and will continue to provide insights into genetic diseases, cancer, and the overall systems of the cell [8]. Therefore, DNA sequencing is a critical tool for biomedical research. Traditional DNA sequencing (Sanger sequencing) is performed by adding chain-terminating nucleotide analogs to a DNA synthesis reaction and then electrophoretically reading the mass or nucleotide added [9]. However, this sequencing is considered low-throughput and cannot access the large amount of information encoded in the human genome. Therefore, the development of more advanced techniques and sensors to detect and sequence DNA is necessary.

A biosensor is a device that can detect a biological substance or analyte by introducing it to a biological recognition element (receptor) and attaching the product to an appropriate transducer where the transducer converts the biological reaction into detectable or measurable signals; e.g., light, current, frequency, etc. There is significant interest in being able to detect and sequence DNA accurately, rapidly, and inexpensively for medical, forensic, and military purposes.

Here, we review the limitations of major techniques for high-throughput DNA sequencing and provide insight into how VLSI implementations can be used for DNA sequencing. Additionally, we propose a novel method to identify DNA sequences using Gate All-Around (GAA) nanowire Metal Oxide Semiconductor Field Effect Transistors (MOSFETs).

## 2. Limitations of Existing Technologies and Need for VLSI Biosensors for Sequencing

There are various high-throughput labeled and label-free DNA sequencing methods and technologies commercially available; some are for short-read sequencing and some are for long-read sequencing. Here, we list some of the limitations of the industry-leading high-throughput DNA sequencing technologies (Table 1 and Table 2). We will be covering some of the second-generation sequencing (SGS) or next-generation sequencing (NGS) methods, which are for short-read sequencing, and the third-generation sequencing for long-read sequencing.

Some limitations of short-read sequencing have overcome by third-generation sequencing approaches at the cost of high error rates. To learn more detail of the advantages and disadvantages of leading DNA sequencing technologies, readers are encouraged to consult [15].

The continuous optimization of CMOS for the purpose of improved computational and memory systems has enabled ultra-scaled devices. These nano-scaled devices also find use in the field of biosensing. There are a number of DNA hybridization detection/sensing techniques available in the literature; however, the use of Field Effect Transistors (FETs) is popular due to the great potential for miniaturization, fast responses, parallel sensing structures, and, most significantly, seamless integration with Complementary Metal Oxide Semiconductor (CMOS) processes. Using VLSI technologies such as CMOS processes, massively parallel integrated circuits can be created with high sensitivity that can be used for DNA sequencing detection. Additionally, a VLSI sensor can incorporate both the embedded analog and digital signal processing needed to increase performance and sensitivity and thus conserve power and reduce overall device size via System-On-Chip (SOC) technology. While DNA sequencing has been done by measuring the ion current through nanopores, incorporating CMOS technology to detect, amplify, and measure this current will improve the sequencing speed, size, and cost [16]. For example, nanopore sequencing by ONT successfully integrated CMOS technology in their DNA sequencing device, the MinION, using an application-specific integrated circuit (ASIC). Although the ASIC SOC was used to control and read out an array of nanopore sensors [17], they were able to utilize the advantages of CMOS technology and became successful in the DNA sequencing industry with a highly portable device. The detailed advantages and implementations of VLSI-enabled sequencing detection are reviewed in [18].

## 3. VLSI Architectures in DNA Detection

A FET sensor can transduce a change in surface potential into an electrical signal. In this section, a general review of the implementation of VLSI devices in DNA hybridization/sequence detection are given. The generalized principle of biological and chemical FET sensors such as Dual Gate FETs (DG-ISFETs), Extended Gate FETs (EGFETs), Floating Gate FETs (FGFETs), and Ion Sensitive FETs (ISFETs) is described in [19].

### 3.1. Electrolyte-Insulator Silicon (EIS) Capacitor

Fritz et al. developed a real-time and selective microfabricated FET sensor, which can detect the increase in the surface charge when the DNA hybridizes on the sensor surface. Their FET sensor is being developed using the Electrolyte-Insulator Silicon (EIS) capacitor structure using a silicon cantilever described in Cooper et al. [20]. Fritz and others chose to measure the capacitance as it requires only one electrical connection to the silicon. They immobilized the probe DNA on a poly-L-lysine (PLL) layer, which is positively charged and allows hybridization at low ionic strength. The reason is that at low ionic strength, this field-effect sensor is very sensitive [21]. The detection limit of their method is 2 nM, and it is mentioned that by using two similar sensors in parallel, it can detect a single base mismatch within a 12-mer oligonucleotide by differential detection techniques.

### 3.2. TFT (Thin Film Transistor)

Estrela et al. developed an Au/SiO_2_/Si MOS diode that is able to detect DNA hybridization by immobilizing the probe on the metal gate of the diode that is in contact with an electrolyte [22]. The immobilized DNA probe affects the interfacial dipole at the gate interface, which eventually influences the voltage drop across the electrochemical layer. This drop adjusts the voltage applied to the gate and changes the CV characteristics of the MOS diode. By observing the changes in C–V curve, it is possible to detect the DNA hybridization. Estrela and others also developed a Poly-Si TFT using the same principle that they used in the MOS diode, to detect DNA hybridization by observing changes in I–V characteristics of the fabricated device. They used a laser recrystallized film with an extended gate.

### 3.3. Different Types of FET’s

#### 3.3.1. MOSFET (Metal Oxide Semiconductor FET)

Kim et al. fabricated a biosensor using 0.5 µm standard CMOS technology that can directly detect the intrinsic charge of the DNA. Using this FET-type DNA charge sensor, they were able to detect the immobilization and hybridization of the DNA probe by sensing the change in the drain current as the intrinsic charge of the immobilized DNA on the gate of the FET, which acts as the applied gate potential [23]. By considering the DNA charge as a Planer electrode charge, a relation between the gate voltage and the DNA charge is achieved (Equation (1)).

(1) $$V_{a}-V_{b}=\;\frac{\rho s}{2}(\frac{d2}{\varepsilon 0\cdot \varepsilon 2}+\frac{d3}{\varepsilon 0\cdot \varepsilon 3})$$

where ρs = surface charge density (Cm^−2^); Ɛ0, Ɛ2, and Ɛ3 = dielectric constant (Fm^−2^) of vacuum, SiO_2_ and Si, respectively; and d2 and d3 = thickness (m) of SiO_2_ and Si respectively.

#### 3.3.2. CMFET (Charge Modulated FET)

Barbaro and others [24] have proposed and simulated a Charge Modulated FET (CMFET) based on floating gate MOS transistors to detect the negatively charged oligonucleotides. The sensor has one control gate and one active area. The probes are immobilized on the surface of an active area surface, which provides the sensing charge. This surface charge will adjust the effective threshold voltage of the transistor predicted by [25]. The control gate is to nullify the unknown charge in the system. The effective threshold voltage can be measured by the drain current for a given control voltage or by measuring the control voltage for a constant drain current. During the hybridization, the charge amount on the sensor surface almost doubles as complementary strands attach to the probe and increase the effective threshold voltage. It is possible to raise the effective threshold voltage higher than the supply voltage by appropriately selecting the control gate voltage and other device parameters so that the device will not conduct. In such a manner, it is possible to detect the DNA hybridization by means of on/off signals of the CMFET.

#### 3.3.3. ISFET (Ion Sensitive FET)

The Ion Sensitive FET (ISFET) was developed from MOSFET technology by removing the gate metal and submerging the silicon dioxide (SiO_2_) along with a reference electrode in an aqueous solution. See [26] for the working principle. In short, using an ISFET, the change in pH due to DNA hybridization can be detected because during the hybridization, hydrogen ions are being released, which reduces the pH of the reaction. An ISFET converts the chemical signals (H^+^) into electrical signals. Various ISFET-based biosensors to detect DNA nucleotides are reviewed in this section.

##### Unmodified CMOS ISFET

Toumazou et al. developed a real-time label-free amplification and detection of nucleic acid full System-on-Chip (SOC) platform using ISFETs, temperature sensors, resistive heating, control circuitry, and a signal processing unit. Unlike other ISFET based sensors, this portable SOC can amplify the reaction signals, detect, and sequence simultaneously. Using this chip, they were able to sequence and detect the cytochrome P450 family from human saliva in 30 min. They have established that using the change in pH, amplification of nucleic acid readout can be done [27]. Identifying SNPs was also possible using this portable battery-operated device. The ISFETs used were fabricated using unmodified CMOS technology, which ensures low cost and scalable devices. Other important research mentioned in the literature based on ISFET to detect DNA sequences are [28,29,30].

##### FGFET (Floating Gate FET)

The Floating Gate FET (FGFET) incorporates two gates—a control gate and a sensing gate—and both are capacitively coupled with a common floating gate so that if any change in potential at either of the two gates occurs, the floating gate potential will adjust accordingly. Jayanta et al. presented a device that can be used to monitor DNA hybridization and manipulate the adsorbed biomolecules at the sensing surface [31]. They used PLL to coat the sensing gate to neutralize the intrinsic hydroxyl charge to facilitate DNA adsorption. During DNA hybridization on the sensing gate, they kept the drain current constant and were monitoring the change in the threshold voltage to detect the hybridization. Jayanta and others also showed that by manipulating the bias voltage and reference voltage, it is possible to manipulate the DNA’s orientation.

##### EGFET (Extended Gate FET)

In an Extended Gate FET (EGFET), the sensing pad is extended off the chip and only the sensing pad is submerged in solution. Thus, this type of sensor separates the wet and the dry environments. Ishige et al. developed an EGFET sensor in which they used an Au (gold) electrode to immobilize the probe on it using a gold–thiol bond. They were able to control the density of the immobilized probe on the gold electrode using a competitive reaction between alkanethiol and the DNA probe. They established a method to measure the density of the immobilized DNA probe and the amount of hybridized DNA probe. By converting the VDS–ID curve into VG using the VG–ID curve at constant VDS = 1 V, they were able to establish that the surface charge density changes due to DNA hybridization [32], and thus, by observing the change in surface charge density, they were able to detect DNA hybridization. This fully electric hybridization detection sensor has a detection limit of 7 fmol, provided that the probe density is 2.6 × 10^12^ molecules/cm^2^ [33].

##### DGFET (Dual Gate FET)

A Dual Gate FET (DGFET) sensor is similar in structure to FGFET sensors with an additional back gate to increase the sensor response. It may be used for DNA detection utilizing a TFT. A Dual-Gate TFT was first introduced in 1981 [34]. Although the use of a DGFET for DNA detection is inadequate, using a DGFET sensor will have a better sensing ability to detect DNA hybridization [35].

Sunil et al. proposed a technique to detect SNPs using an ISFET. They demonstrated the sequencing of a single base through the detection of SNPs, which can be used to sequence new DNA [36]. They monitored the sensor output voltage for a known single nucleotide incorporation and compared it to the output voltage for an unexpected nucleotide incorporation. Observing the difference in voltage level for a complementary nucleotide insertion and a non-complementary nucleotide insertion in a repetitive reaction and recording each procedure four times provides the nature of a single base.

There are various models in the literature that use industry-standard 3D Computer Aided Tools to simulate the biosensing environment all together. Mohammadi and Manavizadeh proposed such a model of ISFET using the Synopsys TCAD tool to detect the surface charge density behavior of the electrolyte and insulator interface and ion concentration according to different pH values [37].

### 3.4. NWFET (Nanowire FET)

The fundamental principle of the semiconductor Nanowire (NW) FET (NWFET) sensor is explained in [38]. Among all the semiconductor NWs (Si, GaN, ZnO, SnO_2_, etc.), silicon NWs have distinct structural and chemical features such as diameters that are at the scale of proteins, their surface to volume ratio is high, and these features enable them to realize high performance FETs for sensitive biomolecular detection. Additionally, it is possible to control the electrical properties and sensitivity of the SiNWs by controlling the doping concentration and the diameter of the NWs [39].

Hahm and Lieber developed a p-type SiNWFET for ultrasensitive and selective real-time DNA and DNA mismatch associated with cystic fibrosis detection [40]. They used Peptide Nucleic Acid (PNA) as the probe instead of ssDNA since PNA has strong and stable binding with DNA with single base specificity [41]. By comparing the change in NW conductance, Hahm and Leiber were able to detect DNA and DNA mismatch at a 10 fM concentration, with the possibility for further improvement in sensitivity. Li et al. from Hewlett-Packard Laboratories developed and fabricated a highly sensitive and sequence-specific SiNW DNA sensor on a Silicon-on-Insulator (SOI) wafer with a sensitivity of 25 pM concentration [42]. They used a 12-mer oligonucleotide probe in their experiment, and sequence-specific DNA detection was obtained by monitoring the change in the conductance of the NW. Ganguly et al. used an organosulfur compound named 3-mercaptopropyl trimethoxysilane (MPTS) on a GaNNW surface to link the probe DNA. By using modified GaNNWs, they observed a wide potential window, as wide as 4.5 V, and a low background current, which added an advantage for the sensitive detection of biomolecules at as low as sub-picomolar concentrations [43]. Ganguly and others were able to detect DNA hybridization by observing the oxidation of the nucleotide through cyclic voltammogram measurements. Ahn et al. demonstrated, for the first time, an independent double-gate FinFET biosensor featuring a silicon nanowire realized on a bulk substrate to detect label-free charged molecules, i.e., polymers and DNA. They were able to demonstrate that threshold voltage (VT) decreases with increasing gate voltage (VG). This VT tunability can be increased by decreasing the width of the nanowire (W_NW_) and thus increasing the sensitivity of the sensor. Instead of immobilizing the DNA vertically via covalent bonds, they demonstrated that immobilizing the DNA flat/horizontally via electrostatic interaction on the sensing surface increases the sensitivity [44]. Using this sensor, Ahn and others were able to detect the BRCA 1 gene for breast cancer detection. Bunimovich et al. also demonstrated that by probing primary DNA through electrostatic adsorption, Debye screening during hybridization can be overcome during DNA hybridization detection [45].

Li et al. fabricated and demonstrated a sequence-specific label-free DNA sensor by monitoring the change in the electronic conductance of a SiNWFET with chemically bonded ssDNA [46]. Chung et al. developed a CAD model to simulate SiNWFET biosensors using the Synopsys 3D TCAD tool. Their simulation model treats the SiNEFET and the electrolyte region together, which includes a more realistic target charge model where the target is described as a charged cube [47]. Here, the charged cube imitates the product charge of a hybridization process between receptor probes and the target molecules.

### 3.5. Carbon Nanotube FET (CNTFET)

Single wall carbon nanotubes (SWNT) are made entirely of surface atoms, and the adsorption of molecules on SWNTs can significantly change the electrical properties of the SWNTs [48]. These changes are measured by observing the conductance or capacitance of the SWNTs, which are usually the gate materials of a FET sensor [49]. To integrate CNTs successfully into an electrical system, controlled deposition at a precise location and proper electrical contacts to metal leads are required. Readers are encouraged to review [50] to know more about the grafting of DNA onto SWCNTs and MWCNTs (Multi wall carbon nanotubes) and various characterization techniques adopted by various researchers, as well as [51] for the development of CNT-based FETs.

Star et al. used synthetic oligonucleotides for selective target DNA sequence detection and that of the SNP of H63D mutation in the HFE gene, which is responsible for genetic hemochromatosis. By observing the change in device conductance, they were able to detect the DNA hybridization sequence and make hemochromatosis SNP discriminations [52].

Chang et al. demonstrated that instead of immobilizing the DNA molecule directly onto the surface of SWNTs, it can be immobilized onto a passivating layer of graphene oxide (GO) covering the SWNTs to avoid the direct attachment of the DNA molecules and keep the intrinsic electrical properties of the SWNTs unchanged [53].

Maehashi et al. developed an aptamer-modified CNTFET to detect immunoglobulin E (IgE). They used 45mer 5’-amino-modified aptamers immobilized on CNT channels and observed the electrical properties of the CNTFET. It was observed that introducing IgE at various concentrations has an effect on the source-drain current [54]. The detection limit of the aptamer-modified CNTFET was 250 pM.

### 3.6. Graphene FET

Hwang et al. developed a fast, portable, and sensitive graphene FET sensor to detect SNPs. This graphene FET consists of two electrodes and a liquid gate chamber. Using this sensor, they were able to detect SNPs in large dsDNA, i.e., 47 nt [55]. They detected a single mismatch by strand displacement-induced resistance, i.e., current change and Dirac point shift in the graphene FET.

### 3.7. CMOS (Complementary Metal Oxide Semiconductor)

Lai et al. developed a sensor based on the method described in [24] for label-free DNA hybridization detection in a CMOS. They have used a layer of alumina as the passivation layer on top of the sensing/active area to ensure the reusability of the device. They were able to detect charges between 10^−14^ and 10^−10^ using their sensor, which includes the usual range of charges being generated during molecular and chemical reactions [56].

Stagni et al. developed and fabricated a fully electronic mix-signal DNA chip with 128 sensor sites including signal processing, and the measurement can be read by connecting it to a computer. Each sensor site has two interdigitated gold electrodes and one integrated measurement circuit. The ssDNA was probed into the surface of the gold electrodes via covalent bonding. Reading the change in capacitance between the sensor site electrode pairs, they were able to detect specific DNA hybridization [57]. Lee et al. also developed a CMOS biosensor for the detection of the H5N1 virus based on capacitance measurements. They were able to detect the target DNA sequence and an SNP up to a detection limit as low as 100 pM [58]. Yusof et al. developed an on-chip microelectrode biosensor based on charge-based capacitance measurement in CMOS technology [59]. They were able to detect DNA by comparing the capacitance measurements among bare, hybridized, and immobilized electrodes.

There are other studies in the literature for label-free DNA detection using CMOS technology based on impedance measurements [60,61]. Readers are encouraged to review [62,63] on CMOS biosensors for DNA hybridization detection.

### 3.8. Nanopore FET

The combination of aa nanopore with a local FET to detect a single molecule that is translocating through the pore is very promising for DNA sequencing. Xie et al. developed a silicon nanowire FET, combining it with a solid-state nanopore to detect the change in electric potential due to the translocation of DNA molecules through the nanopore [64]. Bedell et al. proposed FET-based sensors for sequencing DNA and proteins that are embedded in a nanopore [65]. Their sensors can detect a change in drain current due to the passing of a DNA molecule through the nanopore. Moore et al. developed a nanopore FET in a CAD environment where a modeled nanopore and a gateless MOSFET were embedded in a cell membrane, accounting for the self-consistent Brownian dynamics and Drift-Diffusion techniques in a single simulation domain [66]. They were able to sense the movement of a single ion through the nanopore that can be used as a biosensor.

Yanagi et al. fabricated a side-gated nanopore FET to detect the four types of nucleotides by detecting the changes in the channel current when the DNA translocates through the nanopore [67]. They were also able to see that the current changes in the FET’s channel and the ionic current through the nanopore were in a synchronized pattern when the DNA translocated through the nanopore. They used a two-sided gate to have better control over the channel current. Although they were not able to differentiate the change in the channel current due to individual nucleotides passing through the nanopore, they were able to observe a change in the channel current during the DNA translocation through the nanopore with their device. To detect the individual nucleotides is in the scope of their future research.

The DNA sequence is the order of the bases (nucleotides) within the DNA. If we can detect the order of the nucleotides in a single-stranded DNA of any given length, we can identify the DNA sequence of that length. The way in which we can detect the order of the nucleotides is through the DNA hybridization process, which is the process when a single-strand DNA binds to its complementary DNA strands in a favorable environment. Successfully detecting the nucleotides through the detection of hybridization leads to the identification of the sequence of that DNA. Hence, most of the literature reviewed here is focused on detecting each individual base/nucleotide, which will lead to the detection of the DNA sequence.

## 4. A Novel Method for DNA Sequencing

Due to the charged nature of the nucleotides, it is possible to detect a distinct charge signature for each individual base using a charge-sensitive device such as a MOSFET or other similar technology. Indeed, several groups have been able to detect DNA sequences using a pore on the gate of a MOSFET [68].

**Figure 2:**
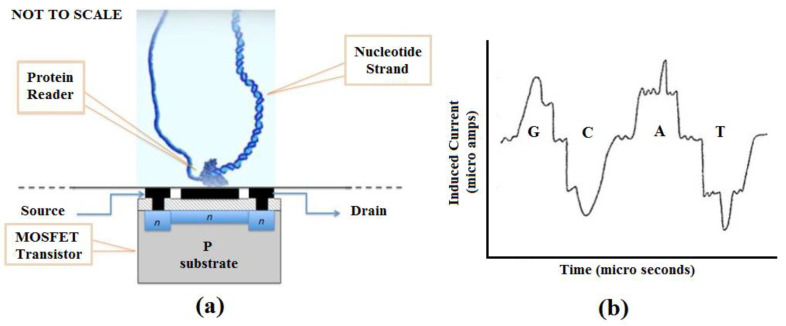
(**a**) Proposed DNA sequencing device; (**b**) Charge representation of nucleotide bases [68].

We propose a nucleotide sequencing device that uses a MOSFET or similar electronic circuit technology to detect the charge signature of nucleotide bases as a target sequence is processed by a helicase, ribosome, or other polymerase motor protein. The color graphic inset figure (Figure 2a) illustrates the basic concept. A motor protein is affixed to a suitable substrate in contact with the gate region of a MOSFET or other charge-sensitive transistor. The motor protein is charged with the target nucleotide strand to be sequenced. The MOSFET is biased to reflect small charge variations as the nucleotide is processed by the protein motor, which acts as a driver to move the nucleotide data tape past the MOSFET gate serving as a read head. The signal produced by the MOSFET is then analyzed to elicit the GCAT sequence present in the nucleotide strand. Figure 2b, though not anticipatory, is from US 20130264204A9 [68] and shows how nucleotides develop unique signatures from induced current. Similar unique signatures will develop from charge variations that occur as the strand is passed across the charge-sensitive gate of the MOSFET. We have simulated a Gate All-Around (GAA) nanowire MOSFET with these properties so that using VLSI technology, we can replicate the device in numbers, create an array, and fabricate the array into a chip (manuscript submitted and under review) [69]. A separate publication describing this technology in detail is in preparation.

## 5. Conclusions

A quick, efficient, and less expensive DNA sequence detection method is required to facilitate various needs such as those of medicine, food, drug screening, the military, and security, as well as others. Since 1958, when the electrochemistry of nucleic acids was first discovered by Palecek, there has been a continual improvement in the development of electrochemical biosensors for DNA analysis using nucleic acids as the sequence recognition element. There are various techniques available in the literature for the direct and indirect detection and labeled or label-free detection of nucleic acids. Among all other methods, electrochemical detection has been attracting researchers due to the feasibility of using VLSI process technologies because of the promise of reduced cost, size, and power consumption, and increased sensitivity, portability, and seamless integration into SOC platforms. The field is wide open as there are no commercially fielded DNA sequencing sensors based on a semiconductor sensor, with the exception of the Ion Torrent Systems, Inc. (now Life Technologies) *Personal Genome Machine*. Lastly, we have proposed a novel technique using nanowire FET technology that could be used to sequence long-strand DNA rapidly with high sensitivity and accuracy.

## Figures and Tables

**Table 1:** Limitations of short-read sequencing technologies.

Sequencing Method	Industry	Limitation
Ion semiconductor	Life Technologies	Homopolymer errors [10]
454 Pyrosequencing	ROCHE	Expensive and homopolymer errors [11]
Sequencing by synthesis (cyclic reversible termination)	Illumina, Qiagen	Expensive equipment [12]
Sequencing by ligation	Applied Biosystems (SOLiD)	Very slow, unable to read palindromic regions [13]

**Table 2:** Limitations of long-read sequencing technologies.

Sequencing Method	Industry	Limitation
Single-molecule real-time	Pacific Bioscience	Higher error rate, Low throughput, Expensive equipment [14]
Nanopore	ONT	Large error rate, Limitation on homopolymer sequencing [15]

## References

[1] Rohs R., Jin X., West S., Joshi R., Honig B., Mann R.S. (2010). Origins of specificity in protein-DNA recognition. Annu. Rev. Biochem..

[2] Lipfert J., Doniach S., Das R., Herschlag D. (2014). Understanding nucleic acid-ion interactions. Annu. Rev. Biochem..

[3] Weiner P.K., Langridge R., Blaney J.M., Schaefer R., Kollman P.A. (1982). Electrostatic potential molecular surfaces. Proc. Natl. Acad. Sci. USA.

[4] Watson J.D., Crick F.H.C. (2003). Reprint: Molecular structure of nucleic acids. Ann. Intern. Med..

[5] Frank-Kamenetskii M.D., Anshelevich V.V., Lukashin A.V. (1987). Polyelectrolyte model of DNA. Uspekhi Fiz. Nauk.

[6] Grosberg A.Y., Nguyen T.T., Shklovskii B.I. (2002). Colloquium: The physics of charge inversion in chemical and biological systems. Rev. Mod. Phys..

[7] Paleček E. (1960). Oscillographic polarography of highly polymerized Deoxyribonucleic acid. Nature.

[8] Skogerboe K.J. (1995). Molecular biology techniques. Anal. Chem..

[9] Men A.E., Wilson P., Siemering K., Forrest S. (2008). Sanger DNA sequencing. Next Generation Genome Sequencing: Towards Personalized Medicine.

[10] Feng W., Zhao S., Xue D., Song F., Li Z., Chen D., He B., Hao Y., Wang Y., Liu Y. (2016). Improving alignment accuracy on homopolymer regions for semiconductor-based sequencing technologies. BMC Genom..

[11] Ronaghi M. (2001). Pyrosequencing sheds light on DNA sequencing. Genome Res..

[12] Kircher M., Kelso J. (2010). High-throughput DNA sequencing - Concepts and limitations. BioEssays.

[13] Huang Y.F., Chen S.C., Chiang Y.S., Chen T.H., Chiu K.P. (2012). Palindromic sequence impedes sequencing-by-ligation mechanism. BMC Syst. Biol..

[14] Rhoads A., Au K.F. (2015). PacBio Sequencing and its applications. Genom. Proteom. Bioinforma.

[15] Goodwin S., McPherson J.D., McCombie W.R. (2016). Coming of age: Ten years of next-generation sequencing technologies. Nat. Rev. Genet..

[16] Magierowski S., Huang Y., Wang C., Ghafar-Zadeh E. (2016). Nanopore-CMOS interfaces for DNA sequencing. Biosensors.

[17] Lu H., Giordano F., Ning Z. (2016). Oxford Nanopore MinION Sequencing and Genome Assembly. Genom. Proteom. Bioinforma.

[18] Singh R.R., Manickam A., Ayazian S., Hassibi A., Shahrjerdi D. VLSI-enabled DNA sequencing arrays. Proceedings of the Midwest Symposium on Circuits and Systems.

[19] Kaisti M. (2017). Detection principles of biological and chemical FET sensors. Biosens. Bioelectron..

[20] Cooper E.B., Fritz J., Wiegand G., Wagner P., Manalis S.R. (2001). Robust microfabricated field-effect sensor for monitoring molecular adsorption in liquids. Appl. Phys. Lett..

[21] Fritz J., Cooper E.B., Gaudet S., Sorger P.K., Manalis S.R. (2002). Electronic detection of DNA by its intrinsic molecular charge. Proc. Natl. Acad. Sci. USA.

[22] Estrela P., Stewart A.G., Migliorato P., Maeda H. Label-free detection of DNA hybridization with Au/SiO2/Si diodes and poly-Si TFTs. Proceedings of the Technical Digest—International Electron Devices Meeting.

[23] Kim D.S., Jeong Y.T., Park H.J., Shin J.K., Choi P., Lee J.H., Lim G. (2004). An FET-type charge sensor for highly sensitive detection of DNA sequence. Biosens. Bioelectron..

[24] Barbaro M., Bonfiglio A., Raffo L. (2006). A charge-modulated FET for detection of biomolecular processes: Conception, modeling, and simulation. IEEE Trans. Electron Devices.

[25] De Lumley-Woodyear T., Campbell C.N., Freeman E., Freeman A., Georgiou G., Heller A. (1999). Rapid amperometric verification of PCR amplification of DNA. Anal. Chem..

[26] Bergveld P. (1970). Short communications: development of an ion-sensitive solid-state device for neurophysiological measurements. IEEE Trans. Biomed. Eng..

[27] Toumazou C., Shepherd L.M., Reed S.C., Chen G.I., Patel A., Garner D.M., Wang C.J.A., Ou C.P., Amin-Desai K., Athanasiou P. (2013). Simultaneous DNA amplification and detection using a pH-sensing semiconductor system. Nat. Methods.

[28] Purushothaman S., Toumazou C., Georgiou J. Towards fast solid state DNA sequencing. Proceedings of the Proceedings—IEEE International Symposium on Circuits and Systems.

[29] Garner D.M., Bai H., Georgiou P., Constandinou T.G., Reed S., Shepherd L.M., Wong W., Lim K.T., Toumazou C. A multichannel DNA SoC for rapid point-of-care gene detection. Proceedings of the Digest of Technical Papers—IEEE International Solid-State Circuits Conference.

[30] Rothberg J.M., Hinz W., Rearick T.M., Schultz J., Mileski W., Davey M., Leamon J.H., Johnson K., Milgrew M.J., Edwards M. (2011). An integrated semiconductor device enabling non-optical genome sequencing. Nature.

[31] Jayant K., Auluck K., Funke M., Anwar S., Phelps J.B., Gordon P.H., Rajwade S.R., Kan E.C. (2013). Programmable ion-sensitive transistor interfaces. II. Biomolecular sensing and manipulation. Phys. Rev. E Stat..

[32] Ishige Y., Shimoda M., Kamahori M. (2006). Immobilization of DNA probes onto gold surface and its application to fully electric detection of DNA hybridization using field-effect transistor sensor. Jpn. J. Appl. Phys..

[33] Kamahori M., Ishige Y., Shimoda M. (2008). Detection of DNA hybridization and extension reactions by an extended-gate field-effect transistor: Characterizations of immobilized DNA-probes and role of applying a superimposed high-frequency voltage onto a reference electrode. Biosens. Bioelectron..

[34] Spijkman M.J., Myny K., Smits E.C.P., Heremans P., Blom P.W.M., De Leeuw D.M. (2011). Dual-gate thin-film transistors, integrated circuits and sensors. Adv. Mater..

[35] Duarte-Guevara C., Lai F.L., Cheng C.W., Reddy B., Salm E., Swaminathan V., Tsui Y.K., Tuan H.C., Kalnitsky A., Liu Y.S. (2014). Enhanced biosensing resolution with foundry fabricated individually addressable dual-gated ISFETs. Anal. Chem..

[36] Purushothaman S., Toumazou C., Ou C.P. (2006). Protons and single nucleotide polymorphism detection: A simple use for the Ion Sensitive Field Effect Transistor. Sens. Actuators B Chem..

[37] Mohammadi E., Manavizadeh N. (2018). An Accurate TCAD-Based Model for ISFET Simulation. IEEE Trans. Electron Devices.

[38] Zhang A., Zheng G., Lieber C. (2016). Nanowires : Building Blocks for Nanoscience and Nanotechnology.

[39] Cui Y., Duan X., Hu J., Lieber C.M. (2000). Doping and electrical transport in silicon nanowires. J. Phys. Chem. B.

[40] Hahm J.I., Lieber C.M. (2004). Direct ultrasensitive electrical detection of DNA and DNA sequence variations using nanowire nanosensors. Nano Lett..

[41] Nielsen P.E., Egholm M., Berg R.H., Buchardt O. (1991). Sequence-selective recognition of DNA by strand displacement with a thymine-substituted polyamide. Science.

[42] Li Z., Chen Y., Li X., Kamins T.I., Nauka K., Williams R.S. (2004). Sequence-specific label-free DNA sensors based on silicon nanowires. Nano Lett..

[43] Ganguly A., Chen C.P., Lai Y.T., Kuo C.C., Hsu C.W., Chen K.H., Chen L.C. (2009). Functionalized GaN nanowire-based electrode for direct label-free voltammetric detection of DNA hybridization. J. Mater. Chem..

[44] Ahn J.H., Kim J.Y., Jung C., Moon D.I., Choi S.J., Kim C.H., Lee K.B., Park H.G., Choi Y.K. CMOS-based biosensors with an independent double-gate FinFET. Proceedings of the Technical Digest—International Electron Devices Meeting, IEDM.

[45] Bunimovich Y.L., Shin Y.S., Yeo W.S., Amori M., Kwong G., Heath J.R. (2006). Quantitative real-time measurements of DNA hybridization with alkylated nonoxidized silicon nanowires in electrolyte solution. J. Am. Chem. Soc..

[46] Li Z., Rajendran B., Kamins T.I., Li X., Chen Y., Williams R.S. (2005). Silicon nanowires for sequence-specific DNA sensing: Device fabrication and simulation. Appl. Phys. A Mater. Sci. Process..

[47] Chung I.Y., Jang H., Lee J., Moon H., Seo S.M., Kim D.H. (2012). Simulation study on discrete charge effects of SiNW biosensors according to bound target position using a 3D TCAD simulator. Nanotechnology.

[48] Snow E.S., Perkins F.K., Houser E.J., Badescu S.C., Reinecke T.L. (2005). Chemical detection with a single-walled carbon nanotube capacitor. Science.

[49] Mehrabani S., Maker A.J., Armani A.M. (2014). Hybrid integrated label-free chemical and biological sensors. Sensors.

[50] Daniel S., Rao T.P., Rao K.S., Rani S.U., Naidu G.R.K., Lee H.Y., Kawai T. (2007). A review of DNA functionalized/grafted carbon nanotubes and their characterization. Sens. Actuators B Chem..

[51] Hu P.A., Zhang J., Li L., Wang Z., O’Neill W., Estrela P. (2010). Carbon nanostructure-based field-effect transistors for label-free chemical/biological sensors. Sensors.

[52] Star A., Tu E., Niemann J., Gabriel J.C.P., Joiner C.S., Valcke C. (2006). Label-free detection of DNA hybridization using carbon nanotube network field-effect transistors. Proc. Natl. Acad. Sci. USA.

[53] Chang J., Mao S., Zhang Y., Cui S., Steeber D.A., Chen J. (2013). Single-walled carbon nanotube field-effect transistors with graphene oxide passivation for fast, sensitive, and selective protein detection. Biosens. Bioelectron..

[54] Maehashi K., Katsura T., Kerman K., Takamura Y., Matsumoto K., Tamiya E. (2007). Label-free protein biosensor based on aptamer-modified carbon nanotube field-effect transistors. Anal. Chem..

[55] Michael T.H., Preston B.L., Joon L., Duyoung C., Alexander H.M., Gennadi G., Ratnesh L. (2016). Highly specific SNP detection using 2D graphene electronics and DNA strand displacement. Proc. Natl. Acad. Sci. USA.

[56] Lai S., Caboni A., Loi D., Barbaro M. (2012). A CMOS biocompatible charge detector for biosensing applications. IEEE Trans. Electron Devices.

[57] Stagni C., Guiducci C., Benini L., Riccò B., Carrara S., Samorí B., Paulus C., Schienle M., Augustyniak M., Thewes R. (2006). CMOS DNA sensor array with integrated A/D conversion based on label-free capacitance measurement. EEE J. Solid-State Circuits.

[58] Lee K.H., Choi S., Lee J.O., Yoon J.B., Cho G.H. CMOS capacitive biosensor with enhanced sensitivity for label-free DNA detection. Proceedings of the Digest of Technical Papers—IEEE International Solid-State Circuits Conference.

[59] Yusof Y., Sugimoto K., Ozawa H., Uno S., Nakazato K. (2010). On-chip microelectrode capacitance measurement for biosensing applications. Jpn. J. Appl. Phys..

[60] Manickam A., Chevalier A., McDermott M., Ellington A.D., Hassibi A. (2010). A CMOS electrochemical impedance spectroscopy (EIS) biosensor array. IEEE Trans. Biomed. Circuits Syst..

[61] Jafari H., Soleymani L., Genov R. (2012). 16-channel CMOS impedance spectroscopy DNA analyzer with dual-slope multiplying ADCs. IEEE Trans. Biomed. Circuits Syst..

[62] Arya S.K., Wong C.C., Jeon Y.J., Bansal T., Park M.K. (2015). Advances in Complementary-Metal-Oxide-Semiconductor-Based Integrated Biosensor Arrays. Chem. Rev..

[63] Lei K.M., Mak P.I., Law M.K., Martins R.P. (2016). CMOS biosensors for: In vitro diagnosis-transducing mechanisms and applications. Lab A Chip.

[64] Xie P., Xiong Q., Fang Y., Qing Q., Lieber C.M. (2011). Local electrical potential detection of DNA by nanowire-nanopore sensors. Nat. Nanotechnol..

[65] Bedell S., D’Emic C., Peng H., Zafar S. (2014). FET Nanopore Sensor. U.S. Patent.

[66] Moore I., Millar C., Roy S., Asenov A. (2012). FET based nano-pore sensing: A 3D simulation study. J. Comput. Electron..

[67] Yanagi I., Oura T., Haga T., Ando M., Yamamoto J., Mine T., Ishida T., Hatano T., Akahori R., Yokoi T. (2016). Side-gated ultrathin-channel nanopore FET sensors. Nanotechnology.

[68] Sauer J., Zeghbroeck J. (2013). Method and Apparatus for Detecting Nucleotides. U.S. Patent.

[69] Islam A. (2020). Mohammad "Gate-All-Around Nanowire MOSFET for DNA Sequencing.". ProQuest Dissertations (Order No. 27961993).

